# Genome-wide identification and characterization profile of phosphatidy ethanolamine-binding protein family genes in carrot

**DOI:** 10.3389/fgene.2022.1047890

**Published:** 2022-11-08

**Authors:** Xing Liu, Donghang Zhao, Chenggang Ou, Weidong Hao, Zhiwei Zhao, Feiyun Zhuang

**Affiliations:** Institute of Vegetables and Flowers, Chinese Academy of Agricultural Sciences, Key Laboratory of Biology and Genetic Improvement of Horticultural Crops, Ministry of Agriculture, Beijing, China

**Keywords:** Daucus carota, PEBP genes, comparative genomics, expression pattern, bolting

## Abstract

Members of the family of Phosphatidy Ethanolamine-Binding Protein (PEBP) have been shown to be key regulators of the transition of plants from vegetative to reproductive phases. Here, a total of 12 PEBP proteins were identified in the carrot (*Daucus carota* L.) genome and classified into FT-like (4), TFL1-like (6), and MFT-like 2) subfamilies, that had different lengths (110–267 aa) and were distributed unevenly across seven chromosomes. Moreover, 13 and 31 PEBP proteins were identified in other two Apiaceae species, celery (*Apium graveolens* L.) and coriander (*Coriandrum sativum* L.). The phylogenetic and evolutionary results of these PEBP family proteins were obtained based on the protein sequences. In the three Apiaceae species, purifying selection was the main evolutionary force, and WGD, segmental duplication, and dispersed duplication have played key roles in the PEBP family expansion. The expression analysis showed that carrot *PEBP* genes exhibited relatively broad expression patterns across various tissues. In the period of bolting to flowering, the carrot FT-like subfamily genes were upregulated as positive regulators, and TFL1-like subfamily genes remained at lower expression levels as inhibitors. More interestingly, the members of carrot FT-like genes had different temporal-spatial expression characteristics, suggesting that they have different regulatory functions in the carrot reproductive phase. In summary, this study contributes to our understanding of the PEBP family proteins and provides a foundation for exploring the mechanism of carrot bolting and flowering for the breeding of cultivars with bolting resistance.

## Introduction

The Phosphatidy Ethanolamine-Binding Protein (*PEBP*) family is an ancient and evolutionarily conserved group that occurs in all three major phylogenetic taxa of prokaryotes, archaea, and eukaryotes, indicating common origin (Hengst et al., 2001; Karlgren et al., 2011). In animals, *PEBP* genes control cell growth and differentiation as kinase and serine protease inhibitors (Lorenz et al., 2003). In plants, *PEBP* genes are involved in many biological processes, particularly in floral transition and plant architecture (Karlgren et al., 2011). Plant *PEBP* genes were originally cloned from mutants with altered inflorescence architecture such as CENTRORADIALIS (CEN) in *Antirrhinum* (Bradley et al., 1996) and TERMINAL FLOWER1 (TFL1) in *Arabidopsis thaliana* (Bradley et al., 1997). To data, many *PEBP* family members have been identified in different plant species, including *Arabidopsis* (Kobayashi et al., 1999; Mimida et al., 2001), tomato (Carmel-Goren et al., 2003), barley (Faure et al., 2007), grapevine (Carmona et al., 2007), maize (Danilevskaya et al., 2008), apple (Kotoda et al., 2010), *Populus* (Mohamed et al., 2010), cotton (Wang et al., 2019), Moso bamboo (Yang et al., 2019) and *Dendrobium huoshanense* (Song C. et al., 2021).

There are three major subfamilies in the *PEBP* family, comprising the FLOWERING LOCUS T (FT)-like proteins, the TFL1-like proteins, and the MOTHER OF FT AND TFL1 (MFT)-like proteins (Danilevskaya et al., 2008; Wang et al., 2019). In *Arabidopsis*, two FT-like genes, *FT* (AT1G65480) and *TSF* (AT4G20370), three TFL1-Like genes, *TFL1* (AT5G03840), *BFT* (AT5G62040), and *ATC* (AT2G27550), and one MFT-like gene, *MFT* (AT1G18100), have been identified. *FT* encodes a floral activator and promotes flowering together with *TSF*, whereas *TFL1*, *BFT*, and *ATC* are antagonists to *FT*/*TSF* (Huang et al., 2012; Ryu et al., 2014; Lee C. et al., 2019; Chen et al., 2021). Additionally, constitutive expression of *MFT* led to slightly early flowering under long days, and *MFT* plays a significant role in seed germination and development. Loss of function mutants showed decreased rates of germination in the presence of ABA (Yoo et al., 2004; Barros-Galvão et al., 2019).

In higher plants, the induction of flowering is a critical developmental decision in the life cycle, representing the transition from vegetative to reproductive growth phases. After extensive research, the plant flowering signal is now relatively well understood, specifically, in *Arabidopsis*, many key genes (e.g., *CO*, *FLC*, *FT*, *SOC1*, *TFL*, and *LFY*) and pathways (e.g., autonomous, photoperiod, and vernalization) have been identified and verified (Bäurle and Dean 2006; Corbesier et al., 2007; Song et al., 2015; Zhang et al., 2020). *FT*, the central flowering regulator, is induced by the circadian clock coordinated gene *CO* and initiates flowering by activating floral promoters such as *SOC1* and floral meristem-identity genes such as *AP1* in meristem when the threshold is passed (Abe et al., 2005; Corbesier et al., 2007; Michaels, 2009; Taoka et al., 2011; Ho and Weigel, 2014). FLC is a MADS-box transcription factor that represses *FT* and *SOC1* in a quantitative manner and plays a central role in the response to vernalization (Lee and Lee, 2010; Sharma et al., 2020). Recently, Zhu et al. (2020) demonstrated that *FT* activation of *LFY* expression is critical for floral fate, and *LFY* as a target under dual opposite transcriptional regulation by *TFL1* and *FT*.

Apiaceae crops are widely cultivated for their unique flavor and medicinal and nutritional value. Carrot (*Daucus carota* L.), celery (*Apium graveolens* L.), and coriander (*Coriandrum sativum* L.) are three representative species that bring significant economic benefits to growers (Ahmad et al., 2019; Maljaei et al., 2019). Carrot is among the top 10 vegetables in terms of global production and is the most significant source of β-carotene in the human diet, and widely distributed in temperate and subtropical regions (Stelmach et al., 2021). However, premature bolting followed by early flowering is the main limiting factor affecting carrot growth and quality, as the xylem quickly lignifies after vernalization, causing a complete loss of commercial value (Wohlfeiler et al., 2019). Mutation of bolting-positive regulators by CRISPR/Cas9 is an important way to obtain bolting-resistant materials. Therefore, an in-depth study of the genes and signal network associated with bolting-resistance in carrot has great significance in application.

To data, only a limited number of *PEBP* genes have been identified in carrot and other Apiaceae crops (Zhan et al., 2017; Liu et al., 2020). Recently, the genomes of carrot (Iorizzo et al., 2016), coriander (Song X. et al., 2020; Song X. M. et al., 2020) and celery (Li M. Y. et al., 2020; Song X. M. et al., 2021) were sequenced and assembled in succession, and these have provided powerful resources for investigating the *PEBP* family genes in the Apiaceae species. In this study, we first identified all potential *PEBP* family genes from the *D. carota* genome, along with *C. sativum* and *A. graveolens* genomes. Their phylogenetic relationships, chromosome locations, structures and motifs, gene duplication events, and collinearity were analyzed. In view of the key roles of *PEBP* genes in flower development, the expression characteristics of carrot *PEBP* genes during bolting and flowering were further analyzed. The results of this study will aid investigation of the detailed molecular and biological functions of *PEBP* members and provides a foundation for breeding carrot cultivars with bolting tolerance.

## Materials and methods

### Identification of *PEBP* family genes in carrot, celery and coriander

The genomic sequence and annotation files of carrot were downloaded from NCBI (https://www.ncbi.nlm.nih.gov/, ASM162521v1). Based on the annotation, the putative full-length protein sequences of all carrot genes were extracted by using TBtools (Chen et al., 2020). Two methods were used to identify PEBP family genes in the carrot genome. First, the Hidden Markov Model (HMM) profiles of the PEBP consensus conserved seed file (PF01161) were downloaded from the Pfam database (http://pfam.xfam.org/) and used as a query to screen the first part of candidate PEBP proteins by HMMER 3.2.1 (e-value < 0.01; Finn et al., 2011). Second, the sequences of six *Arabidopsis* PEBP proteins were downloaded as BLAST queries to identify the second part of PEBP candidate proteins by the BLASTP program. Then, all PEBP candidate proteins from the two part were merged, and further verified by NCBI-CDD (https://www.ncbi.nlm.nih.gov/Structure/cdd/wrpsb.cgi) and SMART (http://smart.embl-heidelberg.de/) and then used for subsequent analysis (Schultz et al., 1998; Marchler-Bauer et al., 2017). The publicly available genomes of celery and coriander were retrieved from Biology to Databases (BIO2DB, http://celerydb.bio2db.com/), and the same method was used to screen out *PEBP* family genes in those species. The ProtParam program (https://web.expasy.org/protparam/) was used to calculate the physical and chemical parameters of PEBP proteins, including theoretical isoelectric point (pI) and molecular weight (Mw). In addition, the subcellular locations of PEBP proteins were predicted using the online ProtComp 9.0 in Softberry (http://linux1.softberry.com/berry.phtml) (Li H. et al., 2020).

### Phylogenetic analyses

Based on multiple sequence alignment of carrot, celery, and coriander PEBP amino acid sequences in Clustal X, the phylogenetic relationships of PEBP proteins were generated by using Molecular Evolutionary Genetics Analysis software package, version 7 (MEGA 7) with the neighbour-joining (NJ) algorithm (Kumar et al., 2016). Bootstrap values from with 1,000 replications were used to assess group support, and the substitution model was the Poisson model. Evolview (http://evolgenius.info/) was used to add colorful visualization plots and to display the phylogenetic tree.

### Chromosomal locations, gene structure and conserved motif analysis

Based on the genome sequences and general feature format (gff) files, the chromosomal locations of each *PEBP* family gene were retrieved and drawn on gene maps using TBtools separately for the three species. Two or more members of the *PEBP* genes arranged closely in the same chromosomal region were identified as one gene cluster. The structures of *PEBP* family genes were obtained online using the Gene Structure Display Server 2.0 (GSDS, http://gsds.cbi.pku.edu.cn/) (Hu et al., 2015). The intron-exon structures and untranslated regions (UTRs) of *PEBP* family genes were also illustrated by GSDS. The conserved motif in full-length PEBP proteins were identified by searching the protein sequence using Multiple Expression motifs for Motif Elicitation (MEME, http://meme-suite.org/), with the following parameters: the maximum number of motifs was 10; the minimum and maximum motif lengths were 4 and 50 amino acids, respectively (Bailey et al., 2009). TBtools was used to visualize the results of gene structure and conserved motif analysis.

### Cis-acting element analysis

The upstream 2,000 bp sequences of *DcPEBP* genes from the transcription start site were extracted from the carrot genome sequences by TBtools. The cis-acting elements were screened and predicted using the PlantCARE database (http://bioinformatics.psb.ugent.be/webtools/plantcare/html/) (Lescot et al., 2002), and TBtools was used to visualize these promoter elements.

### Collinearity, duplication type and evolutionary analysis

MCScanX software (http://chibba.pgml.uga.edu/mcscan2/) was used to obtain the collinearity files between each pair of species (Wang et al., 2012). The duplicate_gene_classifier sub-program was used to identify the duplication type, including singleton, dispersed, proximal, segmental, and whole-genome duplication (WGD). Orthologous and paralogous genes were identified using Orthovenn2 (https://orthovenn2.bioinfotoolkits.net/) (Xu et al., 2019). The non-synonymous (Ka) and synonymous (Ks) values of the orthologous gene pairs were calculated using the calculate_Ka_Ks_pipeline (Qiao et al., 2019) and were used to calculate the Ka/Ks ratio of all homologous gene pairs. The Ka/Ks ratio can generally indicate the nature of selective pressures; if the Ka/Ks ratio is equal to one, this indicates neutral evolution; if the ratio is greater than one, this indicates positive selection, and if the ratio is less than one, this indicates negative/purifying selection (Yang, 2007). For further verification, EasyCodeML (https://github.com/BioEasy/EasyCodeML), a graphical interface for the PAML package, was used to identify positively selected sites in the multiple sequence alignment of orthologous genes (Wang et al., 2009; Ratnaparkhe et al., 2011; Gao et al., 2019). In addition, the approximate divergence time (T) was calculated using the formula T = Ks/2r, where “r” represents the neutral substitution rate (5.2 × 10^–9^ substitutions per synonymous site per year) (Song X. M. et al., 2020).

### Plant materials and gene expression analysis

The annual wild carrot species *D. carota* ssp. *carota* ‘Songzi’ (Ws) supplied by the National Mid-term Genebank of Vegetable Genetic Resources, Chinese Academy of Agricultural Sciences was used for gene expression analysis (Ou et al., 2017). In a greenhouse, Ws was cultivated to complete the whole growth cycle from sowing to harvesting the next generation of seeds. Growth conditions were 28°C with 16 h of light/8 h of dark under artificial light.

For the expression analysis of *PEBP* family genes in different tissues, the root (Ro), hypocotyls (Hy), cotyledon (Co.), vegetative leaf (VL), flower stem (FS), reproductive leaf (RL), bract (Br), inflorescence (In), and mature cremocarp (MC) of Ws were sampled during the different growing stages. To detect the expression pattern of *PEBP* family genes during carrot bolting and flowering, the leaf samples of Ws were taken from bolting to flowering at eight time points: 10 days before bolting (10 DBB), 5 days before bolting (5 DBB), bolting (Bo), 5 days after bolting (5 DAB), 10 days after bolting (10 DAB), 15 days after bolting (15 DAB), flowering (Fl, 20 days after bolting), 5 days after flowering (5 DAF). All samples had three biological repetitions and were frozen in liquid nitrogen and stored at −80°C for RNA extraction.

A FastPure^®^ universal Plant Total RNA Isolation Kit (Vazyme Biotech Co., Nanjing, Jiangsu Province, China) was used to extract the total RNA according to the manufacturer’s protocol. An ND-1000 spectrophotometer (Thermo Fisher Scientific Inc. Wilmington, DE, United States) was used to determine RNA concentration, purity, and integrity, followed by 1% agarose gel electrophoresis. HiScript III All-in-one RT SuperMix (Vazyme Biotech) was used for the generation of first-strand cDNAs, according to the manufacturer’s protocol and diluted to 50 ng/μL for downstream processing. A 20 μL reaction volume contained 10.0 μL of 2 × ChamQ universal SYBR qPCR Master Mix (Vazyme Biotech), 1.0 μL of cDNA, 0.4 μL of each primer (10 μM), and 8.2 μL ddH_2_O. qPCR was carried out in a Bio-Rad CFX96 Real-Time PCR System (Bio-Rad Laboratories, Hercules, CA, United States) with three technical replicates. The reaction protocol was as follows: 95°C for 2 min with one cycle, followed by 45 cycles of 95°C for 10 s, 60°C for 30 s, and 72°C for 20 s. Gene-specific primers were designed using Premier 6 according to the carrot *PEBP* gene sequences. The primer sequences were listed in Supplementary Table S1. The relative gene expression level was analyzed according to the delta-delta Ct (2^−ΔΔCT^) algorithm. The carrot *Tubulin* gene (GenBank ID: XM_017383275) was used as an internal control.

### Subcellular localization and protein modeling analysis

Transient expression in tobacco plants (*Nicotiana benthamiana*) was used to determine the subcellular location of carrot PEBP proteins tagged with green fluorescent protein (GFP). The CDSs sequences of carrot *PEBP* genes fusing to homologous arms were amplified and connected to a pCAMBIA1300-GFP vector by homologous recombination. The recombinant plasmids were transferred into *Agrobacterium tumefaciens* strain GV3101 by chemical transformation. The transformed *Agrobacterium tumefaciens* was inoculated in L-broth supplemented with 50 μg/ml kanamycin, and cultured overnight at 28°C. After precipitation, the *Agrobacterium* pellet was resuspended in infiltration solution (containing 10 mM MgCl_2_, 10 mM MES, and 100 µM acetosyringone) to a desired optical density (OD600 = 0.5). After standing for 2–3 h, the infiltration solution was infiltrated into the abaxial air spaces of *Nicotiana benthamiana* plants (at the age of 4 weeks) with a 1 ml syringe (Voinnet et al., 2000). Two to 4 days after infiltration, the GFP fluorescence was observed by using a Leica SP8 laser confocal microscope (Leica Microsystems Inc. Buffalo Grove, Illinois, United States) at 488 nm spectral emission (matching the GFP), and the empty vector was used as a control. The three-dimensional (3D) structures of *DcPEBP* genes were analyzed using SWISS-MODEL (https://swissmodel.expasy.org/interactive), and PyMOL v2.5 (https://pymol.org/2/) was used to construct visualization plots (Waterhouse et al., 2018).

## Results

### Identification of PEBP family genes

Based on HMMER and BLASTP searches, a total of 12 candidate *PEBP* genes were identified in the whole genome of carrot. The 12 candidate genes were further verified by NCBI-CDD, and the results confirmed that all were *PEBP* family genes with the specific PBP-containing domain (Table 1, Supplementary Table S2). The sequence lengths of the carrot PEBP proteins were ranged from 110 to 267 amino acid (aa) residues, with an average length of 173 aa. *DcTFL1-4* (GenBank accession number: XM_017378851) had the longest ORF length (804 bp), and the molecular weight and theoretical pI of the deduced protein were 30330.33 Da and 9.25, respectively. *DcFT3* (GenBank accession number: XM_017369907) had the shortest ORF length (333 bp), and the molecular weight and theoretical pI were 12481.03 Da and 9.42, respectively. Meanwhile, the subcellular locations of the carrot PEBP proteins were predicted by ProtComp 9.0. All were predicted in the cytoplasm and nucleus. By the same approach, 13 and 31 *PEBP* family genes were also detected in celery and coriander, respectively; these are listed in the Supplementary Table S3. The deduced amino acid sequences of all the 56 PEBP proteins are summarized in Supplementary Table S4.

**TABLE 1:** List of the 12 *PEBP* family genes in carrot.

No.	Gene name	Accession no.^a^	Chromosome no.	ORF length (bp)	Deduced protein			Localization predicted
					Size (aa)	pI	MW (Da)	
1	*DcFT1*	XM_017362136	Chr01	534	177	8.9	20069.78	Cytoplasm, Nucleus
2	*DcFT2*	XM_017361470	Chr07	528	175	7.75	19963.42	Cytoplasm, Nucleus
3	*DcFT3*	XM_017369907	Chr01	333	110	9.42	12481.03	Cytoplasm, Nucleus
4	*DcFT4*	XM_017369916	Chr01	462	153	6.58	17438.76	Cytoplasm, Nucleus
5	*DcTFL1-1*	XM_017378851	Chr02	522	173	9.26	19713.59	Cytoplasm, Nucleus
6	*DcTFL1-2*	XM_017385066	Chr03	525	174	8.64	19510.36	Cytoplasm, Nucleus
7	*DcTFL1-3*	XM_017385406	Chr03	537	178	7.92	20002.84	Cytoplasm, Nucleus
8	*DcTFL1-4*	XM_017385128	Chr03	804	267	9.25	30330.33	Cytoplasm, Nucleus
9	*DcTFL1-5*	XM_017403214	Chr06	528	175	8.58	19639.43	Cytoplasm, Nucleus
10	*DcTFL1-6*	XM_017403215	Chr06	528	175	8.58	19639.43	Cytoplasm, Nucleus
11	*DcMFT1*	XM_017391180	Chr04	447	148	9.37	16571.17	Cytoplasm, Nucleus
12	*DcMFT2*	XM_017366611	Chr08	507	168	5.27	18249.69	Cytoplasm, Nucleus

^a^ The gene ID, in GenBank.

## Phylogenetic analyses and classification of PEBP family proteins

**FIGURE 1:**
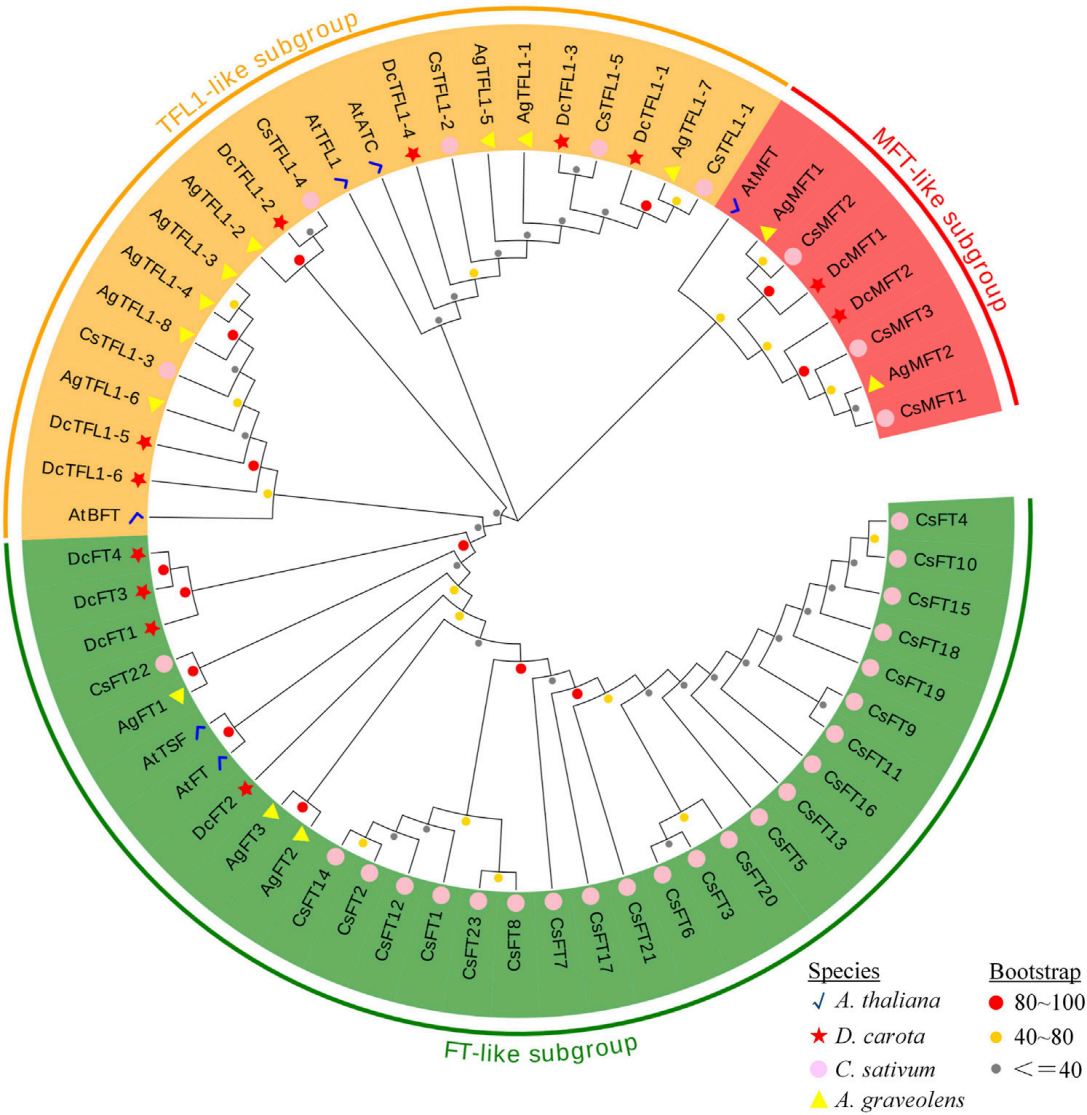
Phylogenetic tree of PEBP proteins from *D. carota*, *A. graveolens*, *C. sativum* and *A. thaliana*. The proteins from each species are labeled with different graphics and colors (red star: *D. carota*, yellow triangle: *A. graveolens*, pink circle: *C. sativum*, blue check: *A. thaliana*). The three subgroups with different colors represent three clades. The circles with different colors at the nodes represent bootstrap percentage values (gray: 0–40, yellow: 41–80, red: 81–100) from 1,000 replications.

To classify the *PEBP* gene family and clarify their evolutionary relationships, MEGA 7 was used to perform a phylogenetic analysis with 62 PEBP amino acid sequences from four species, *A. thaliana*, *D. carota*, *A. graveolens* and *C. sativum*. According to the analysis result, 62 PEBP proteins were clustered into three major groups (Figure 1). This result was consistent with the classification of the *PEBP* family genes in previous studies (Danilevskaya et al., 2008; Wang et al., 2019). In this phylogenetic tree, 30 proteins (four in carrot, three in celery, and 23 in coriander) with 2 *A. thaliana* FT-like proteins (AtFT and AtTSF) were clustered in the same clade, forming the largest FT-like subgroup. In particular, there were more FT-like members in coriander. In addition to CsFT22, other CsFT proteins had rather distant phylogenetic relationships with the DcFT and AgFT proteins. It was speculated that the CsFT proteins may have undergone more duplication events during their evolution. In addition, 19 proteins (six in carrot, eight in celery, and five in coriander) with 3 *A. thaliana* TFL1-like proteins (AtTFL1, AtBFT, and AtATC) were clustered in one subgroup named TFL1-like. The MFT-like subgroup with eight proteins was the smallest clade, comprising two carrot, two celery, and three coriander MFT proteins, and one AtMFT protein. The similar numbers and close relationship of TFL1-like and MFT-like subfamily proteins in the three Apiaceae species indicated that they have undergone similar evolutionary processes.

### Chromosomal locations of PEBP family genes

**FIGURE 2:**
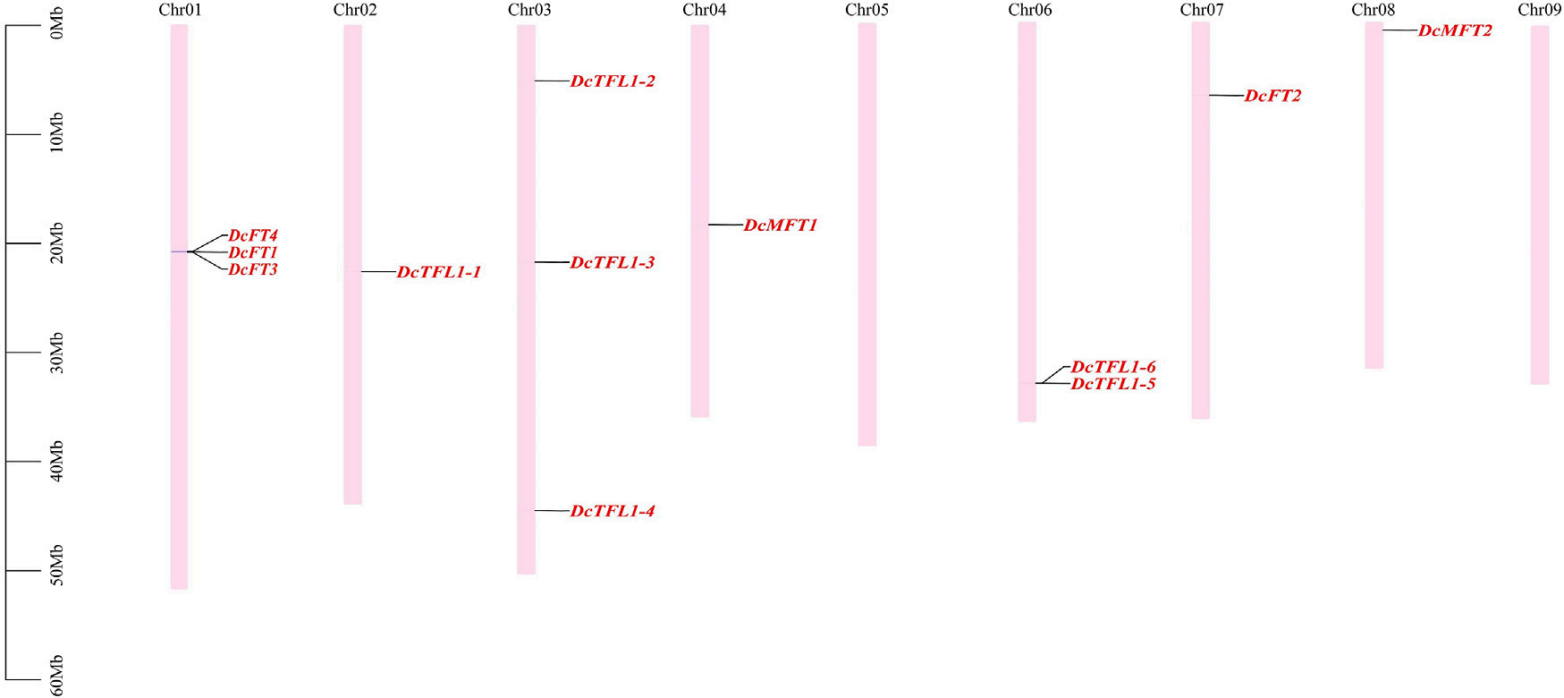
Distribution of *PEBP* family genes on carrot chromosomes. The names of 12 carrot *PEBP* genes are shown to the right of each chromosome. Gene positions and chromosome size can be measured using the scale on the left of the figure in mega bases (bp).

In carrot, 12 *PEBP* family genes were located on seven chromosomes (Chr01, 02, 03, 04, 06, 07, 8), and their distribution on each chromosome was uneven (Figure 2). Chromosomes 01 and 03 contained the highest numbers of *PEBP* genes (three genes), whereas no gene was detected on chromosome 05 or 09. Three carrot FT-like genes (*DcFT1*, *DcFT3*, and *DcFT4*) and two TFL1-like genes (*DcTFL1-5* and *DcTFL1-6*) formed gene clusters on chromosomes 01 and 03, respectively. In celery, 13 *PEBP* family genes were unevenly distributed on eight chromosomes (Chr01, 02, 03, 04, 05, 08, 10, and 11), and chromosome 10 harbored the highest gene number (four genes), but the genes were absent on chromosomes 06, 07, and 09 (Supplementary Figure S1A). Of the 33 coriander *PEBP* genes, 28 were unevenly distributed on 10 chromosomes (Chr01–10). No gene was detected on chromosome 11, and five genes were not anchored to any chromosome (Supplementary Figure S1B). However, the chromosomal distribution of the *PEBP* genes varied among carrot, celery, and coriander, indicative of gain and loss events during WGDs. In previous studies, *DcFT2* on chromosome 7 and *DcFT1* on chromosome 1 had been named (Zhan et al., 2017; Liu et al., 2020). In this study, according to their order on the chromosomes and the subgroup classification result (Figure 1), the other 54 *PEBP* family genes were named one by one in carrot (*DcFT3*–*DcMFT2*), celery (*AgFT1*–*AgMFT2*) and coriander (*CsFT1*–*CsMFT3*) (Table 1, Supplementary Table S3).

### Gene structure, conserved motifs and cis-acting elements analysis

**FIGURE 3:**
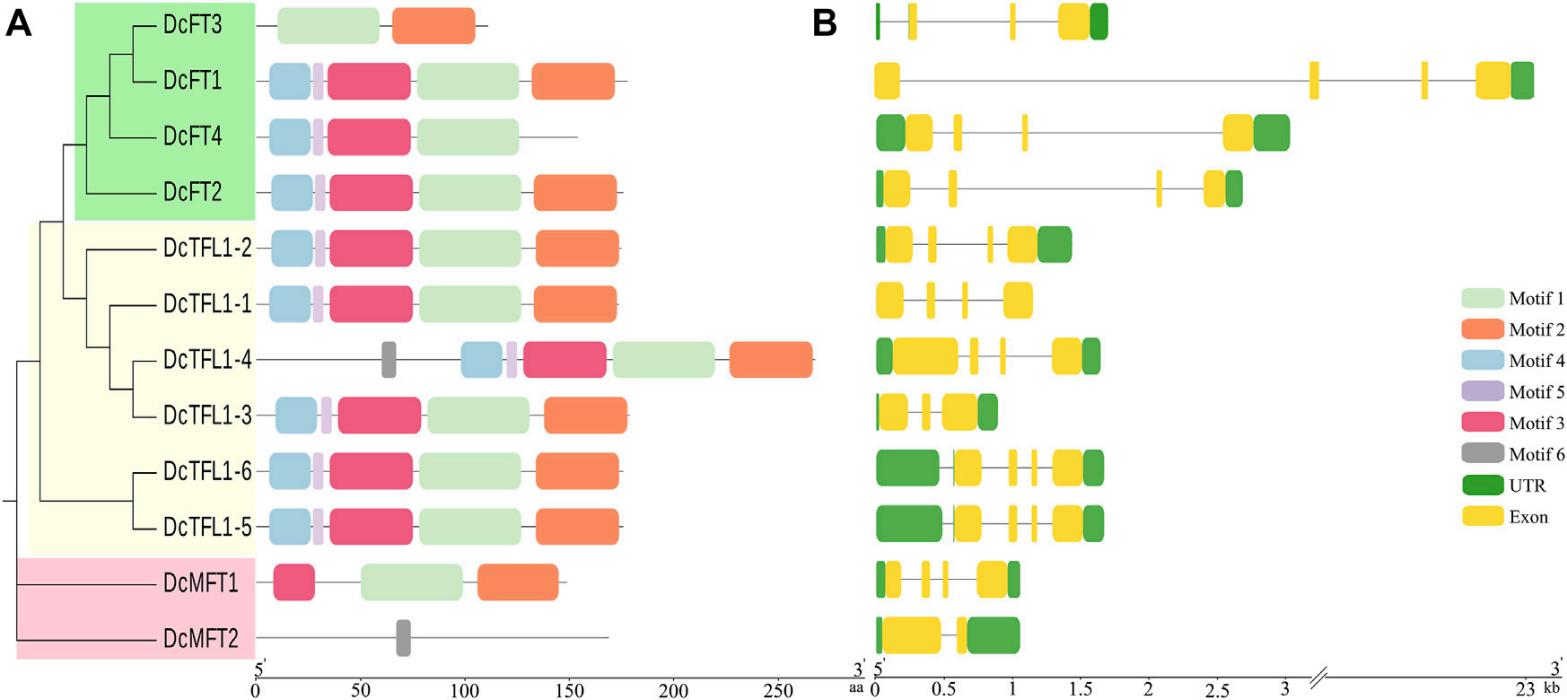
Gene motifs and structures of 12 PEBP genes in carrot. Boxes with different colors indicate conserved motifs, and the lengths of motifs in each protein are shown proportionally **(A)**. UTRs and exons are represented by green and yellow boxes respectively **(B)**.

Six consensus motifs were detected in *PEBP* genes, and the distribution of these conserved motifs was further analyzed in carrot (Figure 3A), celery, and coriander (Supplementary Figure S2A). In carrot, most FT-Like and TFL1-Like genes contained motifs 1–5, especially motif 1 and motif 2. However, *DcMFT2* contained only one motif 6. In celery and coriander, most FT-Like and TFL1-Like genes contained motifs 1–6, whereas motif 6 was absent in all MFT-Like genes. The different motif compositions, especially for MFT-Like genes, may be due to the diversity in the distribution of domains, suggesting that proteins may have different functions in Apiaceae species.

The UTRs, exons, and intron organization of each *PEBP* gene were investigated in the three Apiaceae species (Figure 3B; Supplementary Figure S2B). The majority of the carrot *PEBP* genes contained four exons, whereas *DcFT3*, *DcTFL1-3*, and *DcMFT2* had two or three exons. The length and position of exons and introns of carrot *PEBP* genes were varied. In particularly, *DcFT1* contained a super intron (above 20 Kb). The number of exons in three celery FT-Like genes was consistent (3 exons). However, 17 of 23 *FT* genes had only one exon in coriander. The position and number of exons and introns in genes belonging to the same clade were similar, and this result supported the phylogenetic relationships of *PEBP* family genes.

Furthermore, to identify the responsive elements related to flowering regulation and other biological processes, cis-acting elements analysis was conducted for the 12 *DcPEBP* genes (Supplementary Figure S3). The cis-acting elements responsive to light, phytohormones (auxin, abscisic acid, gibberellin), meristem expression, anaerobic induction, defense and stress, and circadian control were generally detected in the promoter regions of *DcPEBP* genes. However, the number and type of cis-acting elements were distributed differently among genes. These results suggest that *DcPEBP* genes likely contribute to carrot development and several phytohormone signaling pathways, while there has been functional diversification. Interestingly, unlike the other three *DcFT* genes, the light responsive element was not detected in the promoter region of *DcFT2*, indicating that *DcFT2* not directly respond to the changes of photoperiod.

### Collinearity, evolutionary, and duplication type analysis of PEBP family genes

**FIGURE 4:**
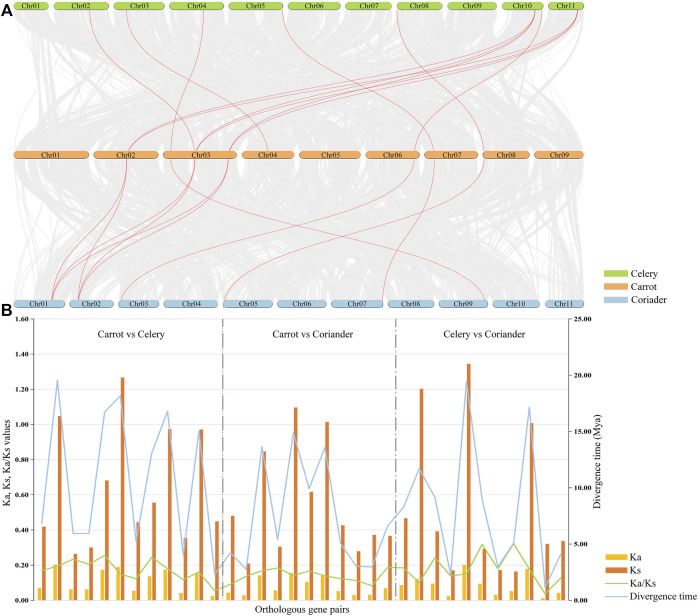
Collinearity analyses of *PEBP* genes among three Apiaceae species. Red lines indicate the intergenomic collinearity **(A)**. Ka/Ks ratios and divergence time of orthologous *PEBP* gene pairs were estimated between any two of carrot, celery and coriander **(B)**.

A total of 34 orthologous gene pairs were identified by collinearity analysis, including 12 between carrot and celery, 11 between carrot and coriander, and 11 between celery and coriander (Figure 4A; Supplementary Table S5). To explore the relationships of *PEBP* genes within species, two paralogous gene pairs were also detected in carrot and celery (Supplementary Table S6). These results indicated that there was a homologous evolutionary relationship between *PEBP* genes in Apiaceae species. To further elucidate the evolutionary dynamics, selection pressure, and divergence time, the Ka and Ks values and Ka/Ks ratios of these orthologous gene pairs were calculated (Figure 4B; Supplementary Table S7). The Ka/Ks ratios of all gene pairs were less than 1.0 in the intergenomic analyses, and no positively selected site was detected by PAML, implying that purifying selection was the main force behind *PEBP* gene evolution in the Apiaceae species. In addition, the divergence time of these orthologous gene pairs was varied from 1.14 to 19.43 million years.

The genome-wide identification of different types of gene duplication was performed, including singleton, dispersed, proximal, segmental, and WGD (Supplementary Table S8). The results showed that 66.7% and 61.5% of *PEBP* genes were derived from WGD or segmental duplication in carrot and celery, respectively, indicating theat WGD or segmental duplication played significant roles in the expansion of carrot and celery *PEBP* family genes. In coriander, 77.4% of *PEBP* genes were derived from dispersed duplication, indicating the key role of dispersed duplication in coriander *PEBP* family genes expansion. No singleton or proximal duplication events were detected in *PEBP* family genes of the three Apiaceae species.

### Expression profiles of PEBP family genes in various carrot tissues

**FIGURE 5:**
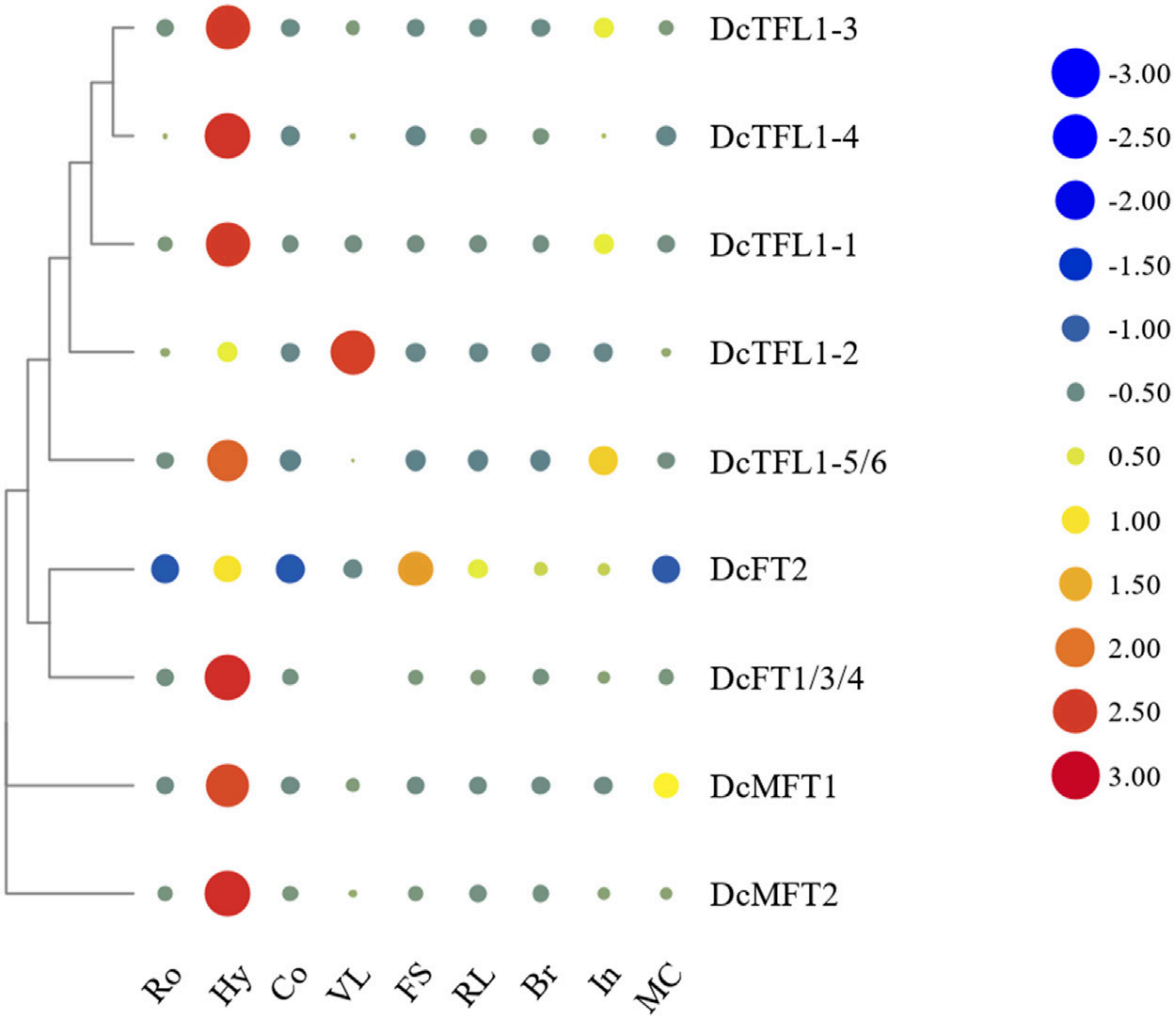
Heat map representation of carrot *PEBP* gene expression levels in various tissues. Ro: root, Hy: hypocotyl, Co. cotyledon, VL: vegetative leaf, FS: flower stem, RL: reproductive leaf, Br: bract, In: inflorescence, MC: mature cremocarp. The color scale with red and blue represent high and low values, respectively; color scale from –3.0 to 3.0.

Because the CDSs of *DcFT1*, *DcFT3*, and *DcFT4* had strong homology (above 96.7%), specific qRT-PCR primers of the three *DcFTs* could not be designed separately. However, one primer pairs DcFT1/3/4 located in the common segment of the three genes was designed to detect their expression levels (Supplementary Table S1). Similarly, one primer pairs DcTFL1-5/6 located in the common segment of *DcTFL1-5* and *DcTFL1-6* was also designed for their expression analysis (Supplementary Table S1). Heat maps of the expression level of carrot *PEBP* genes in various tissues were constructed with horizontal standardization (Figure 5; Supplementary Table S9). Generally, the majority of carrot *PEBP* genes exhibited relatively broad expression patterns across various tissues. *DcFT1/3/4* showed the highest expression level in hypocotyls, while there was almost no expression in roots, cotyledons or bracts. *DcFT2* showed similar expression pattern in roots, hypocotyls and cotyledons; however, higher expression levels were also detected in other tissues. For TFL1-like genes, except for *DcTFL1-2*, the other genes showed the highest expression in hypocotyls, and relatively low expression in cotyledons, flower stems, reproductive leaves and bracts. These *PEBP* genes had different expression patterns in various tissues, even within the same subfamily, suggesting their diverse biological functions. In addition, two MFT-like genes were highly expressed in hypocotyls, indicating their regulatory role in seedling development.

### Expression response of PEBP family genes to bolting and flowering

**FIGURE 6:**
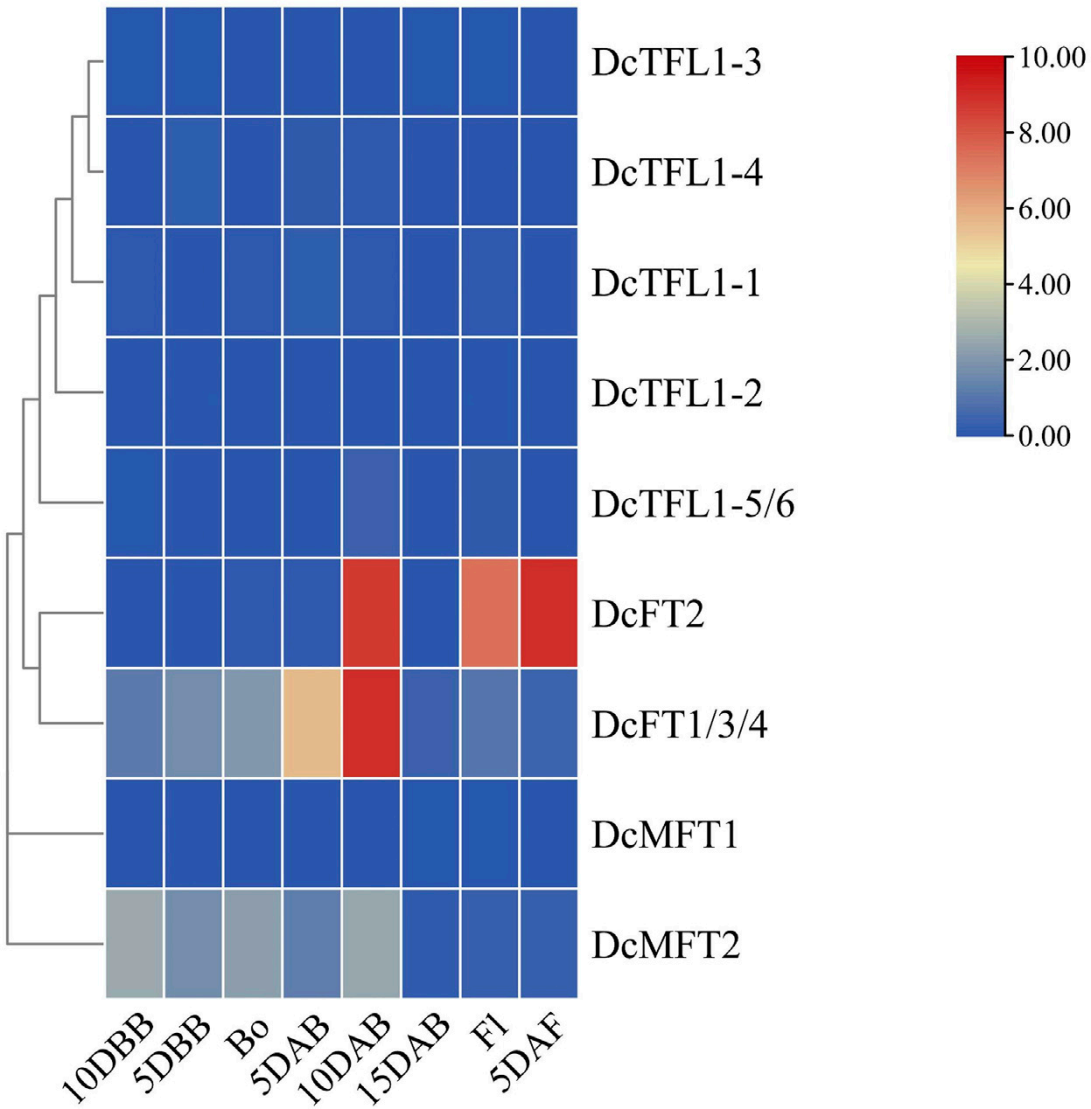
Relative expression of carrot *PEBP* genes from bolting to flowing. DBB: days before bolting, Bo: bolting, DAB: days after bolting, Fl: flowering, DAF: days after flowering. The color scale with red and blue represent high and low values, respectively; color scale from 0 to 10.

Bolting and flowing are important biological processes in plant development, and previous studies have shown that *FT*, *TFL1*, and their orthologs antagonistically regulate these life history traits (Harig et al., 2012; Yu et al., 2020; Zhu et al., 2020; Chen et al., 2022). To evaluate the responses of carrot *PEBP* genes during bolting and flowing, qRT-PCR analysis was conducted at eight time points (10 DBB, 5 DBB, Bo, 5 DAB, 10 DAB, 15 DAB, Fl, and 5 DAF). The expression patterns of carrot *PEBP* genes had significant differences during this period. Several genes (*DcFT1/3/4*, *DcFT2*, *DcMFT2*) showed high expression levels, while the others were basically at a stable low expression levels (Figure 6; Supplementary Table S10). Notably, *DcFT1/3/4* was continuously upregulated from 10DBB to 10 DAB, and the expression level decreased after 10 DAB. In contrast, the expression of *DcFT2* remained low until 10 DAB. The expression patterns of *DcFT1/3/4* and *DcFT2* indicated that both play important role in regulating bolting and flowering, and they had different regulatory functions and temporal-spatial expression characteristics. Similar to other species, all carrot TFL1-like genes showed low expression from bolting to flowering as flowering inhibitors. In addition, two carrot MFT-like genes were differentially expressed, and *DcMFT2* may participate in the bolting and flowering process with high expression level from 10DBB to 10 DAB.

### Subcellular localization and protein modeling analysis of carrot PEBP proteins

**FIGURE 7:**
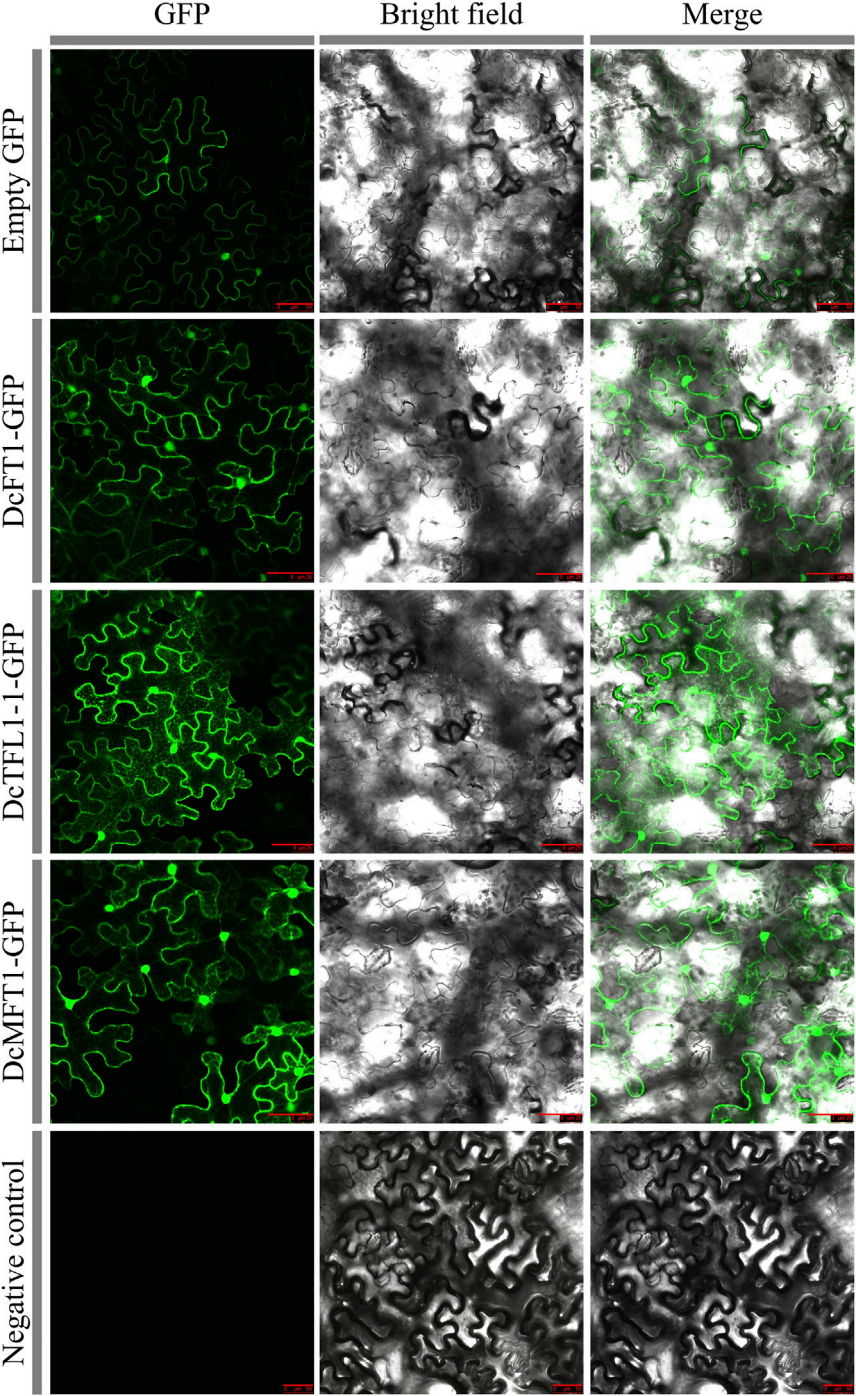
Transient expression of three carrot PEBP proteins in tobacco cells. Bars = 50 μm.

Based on the classification results, DcFT1, DcTFL1-1, and DcMFT1, which participated in three subgroups of carrot PEBP proteins, were selected as representative members to investigate the cellular localization. In the leaves of tobacco plants (*Nicotiana benthamiana*), DcFT1, DcTFL1-1, and DcMFT1 fused to GFP were transiently expressed. By using confocal microscopy, the GFP signals of the three fusion proteins were observed in the peripheral cytoplasm (surrounding the vacuole) and in the nucleus, indicating that there was no restriction to any particular subcellular compartment (Figure 7). The 3D structures of carrot PEBP proteins revealed the existence of multiple alpha helix, beta-sheet and random coil structures (Supplementary Figure S4). The results showed there were different numbers of alpha-helices and beta-sheet among the 3D structures of DcFT, DcTFL1 and DcMFT proteins. Moreover, in the same subfamily, DcFT3 contained two alpha-heliices, while other DcTFL proteins were composed of three alpha-heliices. These differences in the 3D structures may be the reason for functional differences, as DcFT proteins exhibited different expression profiles in the bolting and flowering processes.

## Discussion

Carrot, coriander, and celery, three major members of Apiaceae family, are globally important vegetable crops with rich nutritional value and diverse flavors (Li et al., 2018; Aelenei et al., 2019; Que et al., 2019). Carrot was the first species in the Apiaceae used for genome sequencing, and the genomes of coriander and celery were subsequently sequenced and assembled (Iorizzo et al., 2016; Li M. Y. et al., 2020; Song X. et al., 2020; Song X. M. et al., 2020; Song X. M. et al., 2021). The availability of these genomes provided resources for both fundamental and applied research into Apiaceae species and improved our understanding of the evolutionary and phylogenetic relationships in a group of under-explored plant taxa (Lee H. O. et al., 2019). The estimated genome size of carrot was 473 Mb, with nine chromosomes and 32,113 predicted genes, smaller than that 2,130.29 Mb of coriander and 3553.28 Mb of celery genome with 40,747 and 31,326 predicted genes, resceptively. The genomic differences implied that the genome structure, size and copy number varied considerably among species, and the variations were restricted to repetitive sequences and affected specific gene families involved in different plant physiological processes (Liu X. et al., 2020; Pei et al., 2021). Although the *PEBP* gene family has been reported in many plants, it had not been reported in carrot or other Apiaceae species until now. In this study, we performed genome-wide identification of the *PEBP* family genes in carrot and compared these with the analogus genes in coriander and celery. A total of 12, 13, and 31 *PEBP* family genes were identified in carrot, celery, and coriander genomes, respectively. Interestingly, the gene number and phylogenetic classification of *PEBP* family genes in carrot were similar to those of celery, indicating that the evolutionary rate of *PEBP* family genes was similar between the two species. However, the number of coriander *PEBP* genes in the FT-like subgroup was notably higher than that of the other two Apiaceae species, implying that there were more duplication events of FT-like proteins in coriander.

In this study, we constructed a phylogenetic tree of all *PEBP* family genes in carrot, coriander, and celery, and three distinct subgroups were formed, consistent with previous studies. Gene structure was related to the evolution of various plant species, and protein motifs were the structural elements with specific functional significance (Zhang and Li, 2017; Bu et al., 2021). We found that the motif distribution pattern of most FT-like and TFL1-like genes were similar, indicating that these genes may have conserved functions. However, the motif variation of MFT-like genes was clear, whether in carrot or in coriander and celery, and this may be the reason for the functional diversity of MFT-like genes (Yoo et al., 2004; Xi et al., 2010; Chen and Penfield, 2018; Vaistij et al., 2018). Gene duplication, including tandem duplication, segmental duplication, and WGD are the main types of gene duplication events that serves as the driving forces for gene family expansion in eukaryotes (Freeling, 2009; Jiao et al., 2012; Wang et al., 2013). The WGD or segmental events played an important role in the expansion of the *PEBP* gene family in carrot and celery, while dispersed duplication was the largest attribution to the expansion of coriander *PEBP* gene family. In this study, we identified collinearity blocks among three Apiaceae species by performing interspecies collinearity analyses. Eight carrot *PEBP* genes were found to have orthologous genes in coriander and celery, while five *PEBP* genes had no counterparts in the other two species, suggesting that the five genes were newly duplicated in the carrot genome after the divergence of carrot, coriander and celery. In addition, evolutionary analysis suggested that purifying selection was the primary evolutionary force acting on *PEBP* family genes in Apiaceae species.

It is well known that premature bolting, or early flowering, is an unfavorable factor in carrot production. Therefore, bolting-resistan cultivars ares a major direction in the worldwide carrot breeding effort (Alessandro et al., 2012). However, the regulatory mechanism of the transformation from vegetative growth to sexual reproduction in carrot remains unclear. In a range of plants, *PEBP* family genes, particularly *FT* and *TFL1* subfamily genes, have been reported to participate in flowering differentiation (Zhang et al., 2020; Zhu et al., 2020). In this study, the expression responses of carrot *PEBP* genes to bolting and flowering were identified. The data suggest that carrot *PEBP* genes have different expression responses to bolting and flowering, indicating that they respond specifically to temperature and photoperiod and that they play different roles in flower development. The expression of carrot FT-like subfamily genes varied within the species. We found that *DcFT1/3/4* was involved in the regulation of bolting, while *DcFT2* was responsible for the regulation of flowering. However, more transcriptome analyses should be carried out at different time intervals, so as to clarify the transcripts of *DcFT1*, *DcFT2*, and *DcFT4* separately and to identify the most important bolting regulator in carrot. In the future, based on CRISPR/Cas9, there will be the opportunity to obtain *FT* gene editing lines and thereby breed new bolting-tolerant carrot varieties.

## Conclusion

In summary, 12, 13 and 31 *PEBP* genes were identified in carrot, celery, and coriander genomes, respectively, through genome-wide analysis. The number and length of exons and introns of these *PEBP* genes were varied, and their distributions on each chromosome were uneven. Purifying selection was the main force behind *PEBP* gene evolution in the three Apiaceae species. The expression of the carrot *PEBP* genes was relatively broad in different tissues, and significant differential expression was detected among these genes during bolting and flowing. The results presented in this report contribute to further functional characterization of *PEBP* genes and improve our understanding of the carrot *FT* genes that play key roles in the regulation of bolting and flowering.

## Data Availability

The datasets presented in this study can be found in online repositories. The names of the repository/repositories and accession number(s) can be found in the article/Supplementary Material.
