# Supplementary figures and images for: Genome-wide identification and characterization profile of phosphatidy ethanolamine-binding protein family genes in carrot
Source: Front Genet. 2022 Nov 8;13:1047890. doi: 10.3389/fgene.2022.1047890 (PMC9696379; doi:10.3389/fgene.2022.1047890)

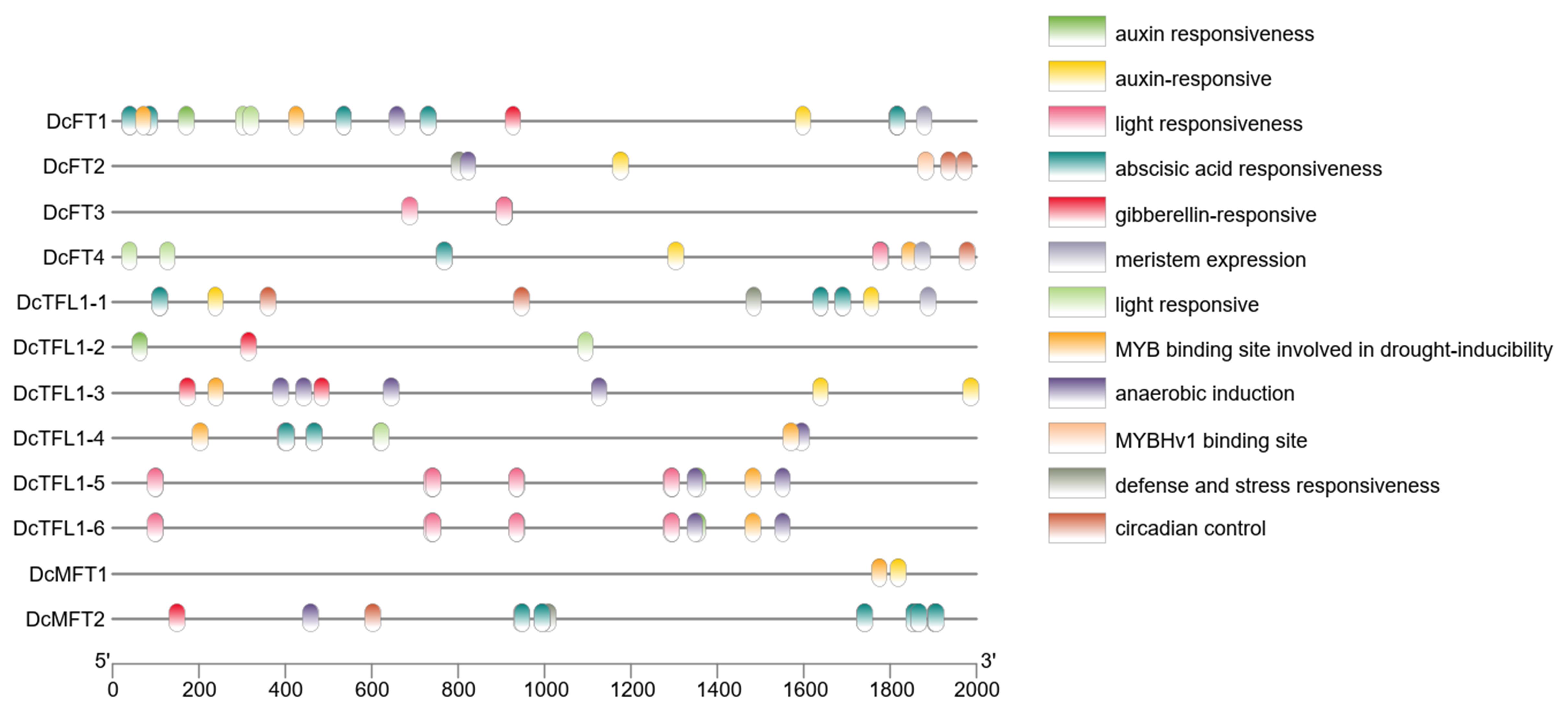

Supplement: Supplementary file 2 [file Image3.JPEG]

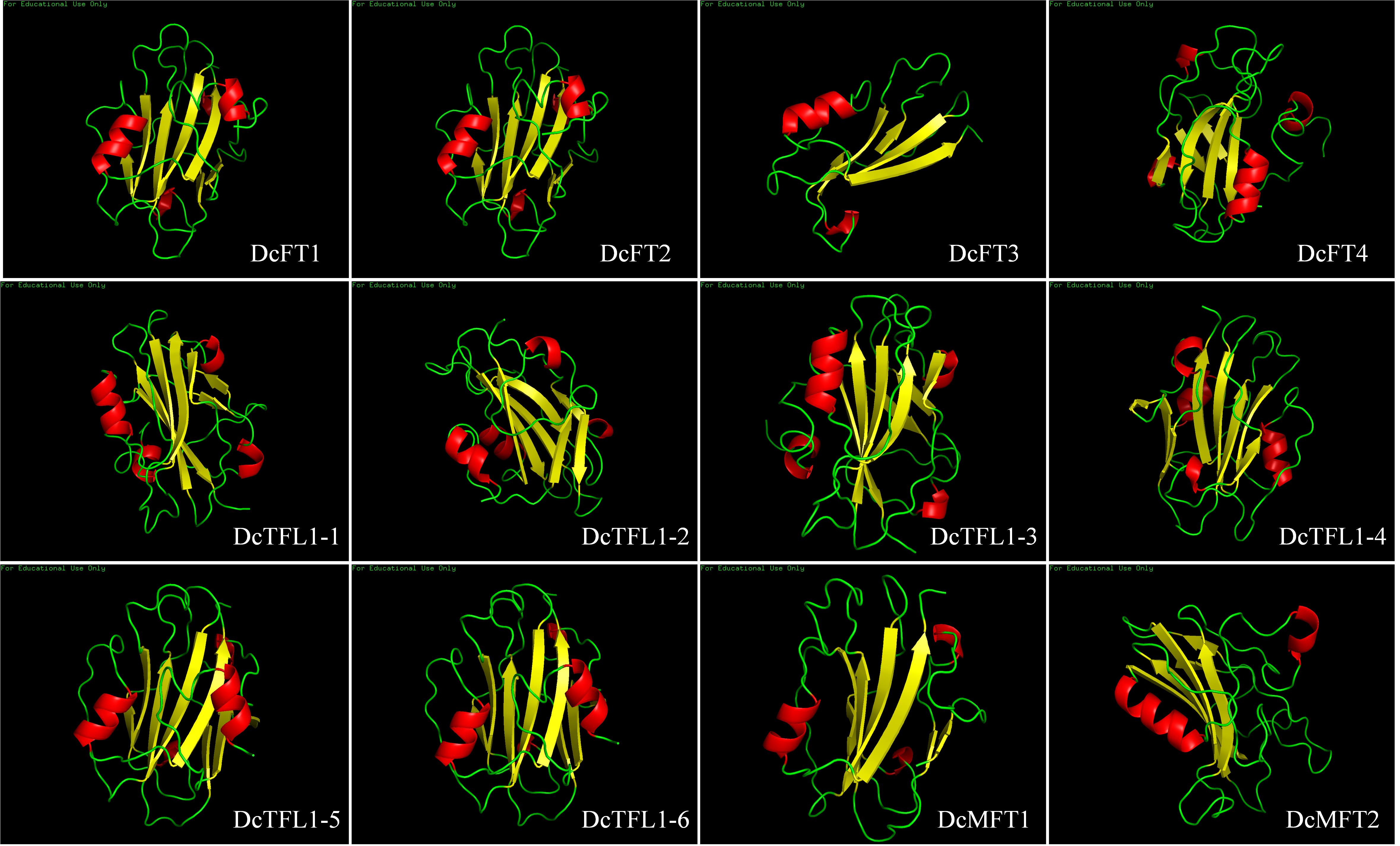

Supplement: Supplementary file 6 [file Image4.JPEG]
